# Arboviruses and pregnancy: are the threats visible or hidden?

**DOI:** 10.1186/s40794-023-00213-w

**Published:** 2024-02-15

**Authors:** Najeh Hcini, Véronique Lambert, Olivier Picone, Jean-Francois Carod, Gabriel Carles, Léo Pomar, Loïc Epelboin, Mathieu Nacher

**Affiliations:** 1 Department of Obstetrics and Gynecology, West French Guiana Hospital Center, Saint-Laurent-du-Maroni, French Guiana; 2 CIC Inserm 1424 and DFR Santé Université Guyane, Cayenne, French Guiana, France; 3 Department of Obstetrics and Gynecology, Hôpital Louis Mourier, Hôpitaux Universitaires Paris Nord Val de Seine, Assistance Publique : Hôpitaux de Paris, Université Paris Diderot, CEDEX, Colombes, France; 4 Department of Biology, West French Guiana Hospital Center, Saint-Laurent-du-Maroni, French Guiana; 5 Materno-Fetal and Obstetrics Research Unit, Department “Woman-Mother-Child”, Lausanne University Hospital, Lausanne, Switzerland; 6 Department of Infectious and Tropical Diseases, Cayenne General Hospital, Cayenne, French Guiana, France; 7 Centre d’Investigation Clinique Antilles Guyane, Inserm CIC1424, Centre Hospitalier de Cayenne, 97300 Cayenne, French Guiana

**Keywords:** Arboviruses, Pregnancy, Fetal outcomes, Maternal, Zika virus, Fever, Dengue fever, Disease outbreaks, Mosquito-borne flavivirus, West Nile virus

## Abstract

Mosquito-borne arboviral diseases are a global concern and can have severe consequences on maternal, neonatal, and child health. Their impact on pregnancy tends to be neglected in developing countries. Despite hundreds of millions of infections, 90% pregnancies being exposed, scientific data on pregnant women is poor and sometimes non-existent. Recently and since the 2016 Zika virus outbreak, there has been a newfound interest in these diseases. Through various neuropathogenic, visceral, placental, and teratogenic mechanisms, these arbovirus infections can lead to fetal losses, obstetrical complications, and a wide range of congenital abnormalities, resulting in long-term neurological and sensory impairments. Climate change, growing urbanization, worldwide interconnectivity, and ease of mobility allow arboviruses to spread to other territories and impact populations that had never been in contact with these emerging agents before. Pregnant travelers are also at risk of infection with potential subsequent complications. Beyond that, these pathologies show the inequalities of access to care on a global scale in a context of demographic growth and increasing urbanization. It is essential to promote research, diagnostic tools, treatments, and vaccine development to address this emerging threat.

**Background **The vulnerability of pregnant women and fetuses to emergent and re-emergent pathogens has been notably illustrated by the outbreaks of Zika virus. Our comprehension of the complete scope and consequences of these infections during pregnancy remains limited, particularly among those involved in perinatal healthcare, such as obstetricians and midwives. This review aims to provide the latest information and recommendations regarding the various risks, management, and prevention for pregnant women exposed to arboviral infections.

## Introduction

Recent COVID-19 and Zika virus (ZIKV) epidemics, both declared as global health emergencies by the World Health Organization (WHO), highlight that pregnant women and fetuses are a public of concern for emerging infections because of their immune vulnerability. Among emergent infectious agents, arbovirus infections are gaining ground. More than 110 arbovirus species are pathogenic to humans [1]. However, more than 500 arboviruses have been recognized worldwide which may represent less than 1% of the total [1]. While infections are often asymptomatic or minimally symptomatic in the healthy adult population, some arboviruses are of great concern in pregnant women and fetuses [2–4]. The global health impact of these infections includes acute mortality, post-infectious persistent disabling diseases, long-term ocular and neurologic morbidity, and congenital diseases. Thus, arboviruses represent major international public-health concerns that have led the WHO to announce on March 22, 2022, the launch of the Global Arbovirus Initiative [5].

There is no consensus on management of arbovirus infection during pregnancy, except for ZIKV. The existing evidence is predominantly derived from observational reports, which occasionally present conflicting findings. The recent epidemic caused by ZIKV and the demonstration of vertical transmission of Tonate virus (TONV) [6] has called attention to the disease burden caused by arboviral infection in pregnant women and their fetuses. In fact, severity of lesions is directly related to gestational age. During the first trimester, embryogenesis and neurogenesis are periods of particular risk of severe birth defects and fetal losses [2, 7]. Late infections are more likely to cross the placenta, which is more permeable in late pregnancy, to infect the fetus, but the fetus is no longer at risk of embryopathy unlike early infections [8]. In exposed areas, adverse outcomes in pregnancy may be aggravated by other associated factors such fragility of the health care system, social deprivation, anemia, insecticides, and heavy metal poisoning [9]. These factors, along with nutritional deficiencies, may exacerbate the risk of perinatal infection and prematurity.

The aim of this narrative review is to provide the most up-to-date information and recommendations for healthcare providers, including obstetricians, maternal-fetal medicine specialists, and midwives who are providing care for pregnant women that are exposed to these emerging pathogens.

## Epidemiology

Most of the cases of arbovirus infections in pregnant women in the world concerned the two families *Flaviviridae* and *Togaviridae*. It is responsible for an increasing number of human outbreaks of neuroinvasive and visceral diseases. The *Flavivirus* genus of the *Flaviviridae* family include Dengue virus (DENV; species name *Orthoflavivirus denguei*), West Nile virus (WNV; species name *Orthoflavivirus nilense*), Zika virus (ZIKV; species name *Orthoflavivirus zikaense*), and yellow fever virus (YFV; species name *Orthoflavivirus flavi*). The *Alphavirus* genus of the *Togaviridae* family includes Venezuelan equine encephalitis virus (VEEV) and Chikungunya virus (CHIKV). Almost 90% of pregnancies worldwide can occur in areas exposed to these viruses and an estimated 4 billion people live in areas at risk for DENV transmission alone [10]. Most infections have been reported in tropical and subtropical regions. Furthermore, The burden of mosquito-borne arboviruses, as for other zoonoses, continues to grow at a global scale [11]. DENV, CHIKV, and YFV are endemic in many tropical areas in South America and Africa and result in frequent underreported outbreaks. CHIKV causes major epidemic outbreaks in Africa, Asia, countries bordering the Indian Ocean, and more recently the Americas. WNV is now endemic in Africa, Asia, Australia, the Middle East, Europe, and the Unites States [12]. The first major recognized outbreak since 1973 of VEEV occurred in Venezuela and Colombia in 1995 and involved an estimated 75,000 to 100,000 people [13]. However, the number of infected pregnant women during outbreaks remains unknown.

During the 2015–2016 ZIKV epidemic, once thought to be restricted geographically, the virus spread to more than 87 countries, most of which were low-income [7]. The actual number of cases is thought to be much higher than the 500,000 that were reported at the peak of the pandemic in 2016 due to the asymptomatic or pauci-symptomatic nature of the infection and lack of diagnostic tools [7]. Serological surveys confirm a significant cumulative risk of arboviral infections among young and active pregnant women residing in epidemic-prone regions. It was estimated at 21 to 63% for Zika virus infection in pregnant women, 26% for CHIKV during the 2005–2006 epidemic outbreak in Mayotte [14], and 2.8% during the 2008–09 DENV epidemic in Brazil [15]. The rapid occurrence of numerous infections in a condensed period can lead to the rapid overburdening of the healthcare system, impacting both human and material resources. This unpredictable phenomenon explains in part the difficulties in managing these infections and setting up good quality studies on the subject.

Co-infection with two or more arboviruses are possible. In a study by Carrillo-Hernández et al. between August 2015 and April 2016, among 157 patients with febrile syndrome consistent with DENV infection, the prevalence of DENV/CHIKV, DENV/ZIKV, and CHIKV/ZIKV co-infection using PCR testing was 7.64, 6.37, and 5.10%, respectively [16]. Populations living in tropical and subtropical areas are continuously exposed to insect bites of arbovirus vectors, with sustained immunological responses that can play both a protective role or a risk factor in the event of subsequent exposure [17].

Arboviral infections like WNV and Rift Valley fever virus (RVFV) exert impacts on both human and animal populations, leading to considerable economic and nutritional consequences [18, 19]. Over the past two decades, a dramatic expansion in the number of cases and in geographical distribution of arboviral infection has been documented. For example, there has been a genetic adaptation of CHIKV and DENV to *Aedes albopictus* [20], a mosquito species that thrives in temperate regions. In fact, a large number of autochthonous cases of DENV and CHIKV occurred in Europe, the Middle East, and Oceania [21, 22] and, therefore, increases risk of local outbreaks. *Ae. albopictus* is spreading geographically [21].

The increasing number of pregnant travelers, the mobility of the world population, and the ecological suitability of the vector *Ae. albopictus* in many regions of Europe are contributing factors to potential transmission of arboviral infections.

## Mode of transmission

### Vector-borne transmission

The primary mode of transmission of arboviruses is vectorial. It is maintained by urban and forest cycles, with varying degrees of determination, and involves arthropod vectors such as mosquitoes, sandflies, ticks, and human or vertebrate hosts. In the urban cycle, mosquitoes of *Aedes* species, especially *Ae. aegypti* and *Ae. albopictus*, constitute the main vectors of arboviruses in the world. In addition to these two species, arboviral infections can also be transmitted by *Ae. hensilii*, and *Ae. polynesiensis*. *Culex* mosquito species are the principal vectors that spread WNV, St. Louis encephalitis virus (SLEV; species name *Orthoflavivirus louisense),* and Japanese encephalitis virus (JEV; species name *Orthoflavivirus japonicum*) in tropical and temperate regions.

After the bite from an infected mosquito, the virus passes from the host to a new host. Most of the alphaviruses are transmitted between mosquitoes and vertebrates [6]. They are able to replicate in a wide number of hosts, including mammals, birds, reptiles, amphibians, and arthropods [23]. Most arboviruses first had enzootic circulation between nonhuman primates and sylvatic mosquitoes before expanding to include transmission by humans. Once attached to the cell, viral particles are internalized by endocytosis which is followed by viral replication within the cytosol of the infected cell.

### Sexual transmission

Evidence of sexual transmission of ZIKV has been established [24] and was suspected for Crimean-Congo hemorrhagic fever virus (CCHF) and DENV [25]. Furthermore, many arboviruses have been isolated from or indirectly detected in the urogenital tract and sexual secretions of their vertebrate hosts including humans [25]. However, identification of arboviruses in the genital tract does not necessarily mean that it can be transmitted during sexual intercourse.

ZIKV sexual transmission constitutes an important turning point in our knowledge about arbovirus infections and their propagation. ZIKV is more likely transmitted from men to women than from women to men through vaginal, oral, or anal intercourse [26]. The window of sexual transmission remains uncertain, and it appears possible several weeks after the appearance of symptoms. This led the CDC during the ZIKV epidemic to recommend pregnant women and their partners who have travelled to or lived in an area with risk of ZIKV infection to use condoms when practicing vaginal, anal, or oral sex or abstain from sex for the duration of the pregnancy [27]. However, the role of sexual transmission in the onset and/or transmission of arboviruses like ZIKV remains unclear. In endemic areas, the proportion of each mode of transmission of infection is not known and difficult to evaluate due to continuous exposure to mosquito bites.

ZIKV RNA has been detected in semen 6 months after symptom onset [28]. The implication for this finding and similar data is that reproductive tissue donors are recommended to wait at least 6 months after infection before giving a specimen [29]. Finally, investigation on the possibility of sexual transmission of arboviruses is warranted. Although not an arbovirus, the recent discovery that Ebola virus can be transmitted sexually, even long after acute infection, has led to concern that this could be a source for the emergence of new epidemic outbreaks [30].

### Transfusion, bone marrow or organ transplantation, and nosocomial transmission

The risk for transfusion transmission of arboviruses is due to asymptomatic infections, capacity of testing, and sometimes extremely high incidence of arboviral infections. Transmission of ZIKV by blood transfusion is possible. There are several studies which indicate that transmission through blood transfusions of DENV and CHIKV in epidemic areas is possible [31]. Within regions of heightened risk, the prevalence of DENV surpasses 5% among blood donors, while CHIKV and ZIKV prevalence exceeds 1 and 2%, respectively [32], giving rise to uncertainties concerning the potential impact such transfusions on pregnancy courses. In DENV infection, organ transplantation is already proved to be an atypical mode of transmission [33]. There are case reports describing ZIKV infection in organ transplant recipients [34]. WNV can be transmitted by blood transfusion and organ transplantation [35]. Nosocomial transmission of DENV after needlestick injury has been reported [36, 37]. However, data on transmission of ZIKV via transplantation, needlestick injury, and laboratory work is inconclusive [38] since it is still difficult to rule out vector-borne transmission in recipients.

### Mother-to-child transmission (MTCT) of arboviruses

MTCT includes transplacental transmission, intrapartum contact with body fluids and mucous membranes during the passage through the genital tract, postpartum contact, and transmission from breastfeeding. The risk of MTCT varies significantly among different arboviruses (Table 1). Gestational age at the time of infection and the vertical transmission ability of different arboviruses are a key element that will define the impact of the infection on the outcome of the pregnancy. The first trimester of pregnancy is a critical period for teratogenic arboviruses like ZIKV [39], while the perinatal period is significant for arboviruses with risk of perinatal transmission, such as DENG, and CHIKV and YFV [40, 41]. The rate of prenatal transmission can reach 50% for CHIKV in mothers with intrapartum viremia [42] and almost 30% for ZIKV vertical transmission during pregnancy [3]. On the other hand, in endemic areas, many arboviral infections, such as dengue and chikungunya, are common in first and second trimester of pregnancy and typically do not result in significant consequences. Many questions remain unanswered, including issues like the tropism of some arboviruses for trophoblast and placenta, the duration between maternal infection and amniotic transmission of the virus, the correlation between placental and fetal infection, the impact of maternal viral load on transplacental transmission, and the potential for fetal infection without clinical consequences.

**Table 1:** Summary of the current state of knowledge of mother-to-child transmission of arboviruses

	Vertical transmission during pregnancy	Perinatal transmission	Breastfeeding
**ZIKV**	Documented in first, second trimester, and third trimester	rare	Not documented
**DENV**	Documented: Low incidence	Documented	Not documented
**CHIKV**	Documented: Low incidence	Documented with intrapartum viremia	Not documented
**VEEV**	Documented: Rate unknown	No data	Not documented
**OROV**	No data	No data	No data
**YFV**	No data	Documented: Rate unknown	Not documented
**WNV**	Documented rare	Suspected	Not documented
**RVFV**	Documented: Unknown incidence	Documented: Rare	Not documented
**JEV**	Documented: Rare	Documented: Rare	No data

Vertical transmission primarily refers to transmission during pregnancy, while perinatal transmission encompasses the period around the time of childbirth and may include labor, delivery, and breastfeeding

*CHIKV* Chikungunya virus, *DENV* Dengue virus, *JEV* Japanese encephalitis virus, *OROV* Oropouche orthobunyavirus, *RVFV* Rift Valley fever virus, *VEEV* Venezuelan equine encephalitis virus, *WNV* West Nile virus, *YFV* Yellow fever virus, *ZIKV* Zika virus

#### Transplacental transmission

Transplacental transmission depends on the capacity of arboviruses to cross the placenta and the term of pregnancy at the time of infection. The placenta constitutes the principal physical and immunologic barrier between the maternal and fetal compartments. The detection of pathogens in the placenta does not necessarily equate to fetal infection. Once the virus breaches the placental barrier and enters the fetal compartment, fetal damage is contingent on the virus’s affinity for fetal tissues, its teratogenic effects, the timing during embryogenesis, and the severity of viral injury. To reach the fetal brain, neuroteratogenic viruses must breach the placenta and fetal blood–brain barrier. However, the mechanism by which arboviruses cross the blood–brain barrier remains largely unknown. Once the virus reaches the target organ and beyond the initial viral cytopathic effects, the inflammatory reaction within fetal tissue of the host can also cause injury and developmental defects. Transplacental transmission is well documented in ZIKV, VEEV [39, 43] and rarely described in WNV, DENV and CHIKV infections. During the prenatal period, ultrasound scans may reveal visible damage that reflects both the direct and indirect effects of the infection. The teratogenic effect of mosquito-borne arboviral diseases is only documented in ZIKV and VEEV, but other rare arboviral infections remain poorly studied in this regard.

CHIKV and YFV are not able to infect the placenta despite high maternal viraemia. Vertical transmission of CHIKV, DENV and WNV remains rare [44]. Some viral arboviruses such DENV and ZIKV can infect placenta and induce pathological alterations, including upregulation of pro-inflammatory cytokines such as interleukin 6, interleukin 8, and TNF-5. These mechanisms can lead to miscarriage or stillbirth [45].

#### Perinatal transmission

This mode of transmission is well documented in cases of YFV, CHIKV, and DENV infection but remains rare for ZIKV infection. In CHIKV infection, intrapartum transmission ranges from 27.7 and 48.3%, with a strong relationship to maternal viremia coinciding with the time of birth [46]. In the study by Basurko et al., the estimated neonatal infection rate in cases of maternal dengue occurring near delivery was 56.2% [47]. There was no correlation between the presence or severity of maternal symptoms and neonatal outcomes. However, the mode of delivery (cesarean section or natural delivery) did not appear to impact the incidence of MTCT after CHIKV infection [8]. Perinatal transmission rates for other arboviruses, such as YFV, WNV, and RVFV, have not been investigated.

#### Breastfeeding

Few studies have focused on MTCT during breastfeeding. The studies that have examined this issue have pointed to the vaccine strain of YFV, strongly highlighted for ZIKV, and suggested potential involvement for CHIKV, DENV, and WNV [48]. MTCT of ZIKV through by Breastfeeding was demonstrated in a mouse model [49]. ZIKV and WNV have been isolated from human milk, but milk-borne transmission has not been confirmed [50, 51]. Unlike in other viral infections where the virus is concentrated in breast milk, such as in HTLV-I, CMV, and HIV [52], transmission via breastfeeding seems to be marginal or non-existent for arboviruses. Considering the potential risk compared with the innumerable benefits of breast milk, continuation of breastfeeding is in the best interest of the infant and mother. In cases of maternal ZIKV infection, the WHO still recommends breastfeeding. Further studies are required to assess the benefit of breastfeeding versus the risk of neonatal infection in endemic areas.

## Clinical presentation in infected pregnant women and neonates

Depending on cellular and tissue tropism of each arbovirus, clinical manifestation and diseases can be either neurotropic, visceral, and/or congenital [53]. The majority of arboviral infections lead to either an asymptomatic or non-specific mild illness. After a short incubation period of a few days, arboviral disease typically presents as a mild flu-like or algo-eruptive syndrome with fever, maculopapular rash, arthralgia, myalgia, weakness, headache, and a maculopapulous eruption [54]. Some subtle signs allow differentiation between arboviruses. Joint pain is a characteristic symptom of CHIKV infection, while an intense headache is typical of DENV infection. Bleeding can complicate YFV infection or severe dengue. Conjunctival hyperemia is more frequent in ZIKV infections. These symptoms are not necessarily associated with the severe form of the disease [55, 56]. Pregnancy is generally not associated with more frequent or severe maternal complications such as in ZIKV and CHIKV infection [41, 57] and globally the clinical features are similar in pregnant compared to non-pregnant women [7]. Mean duration of joint pain was reported to be shorter among women infected during pregnancy compared with infections outside of pregnancy [58]. During pregnancy, biological abnormalities that include lymphopenia, thrombocytopenia, and increased serum transaminase levels can mimic a pregnancy-associated complication such as HELLP (hemolysis, elevated liver enzymes, and low platelets) syndrome and therefore can delay diagnosis. Serious presentations can range from hemorrhagic fever (e.g., DENV, YFV, and RVFV), shock syndromes (e.g., DENV), and brain affection like meningoencephalitis and their frequency varies widely between arboviruses. Some arboviruses such as YFV, JEV, and RVFV can lead to serious neurological complications and life-threatening diseases such as mental confusion, meningoencephalitis, myelitis, acute flaccid paralysis, and Guillain-Barré syndrome [53–55, 59–62]. Acute motor weakness, encephalopathy, seizures, and myoclonus were reported as neurological manifestations of DENV infection [63]. Furthermore, some arboviruses such WNV, DENV, ZIKV, CHIKV, and RVFV have been associated with an array of transient ocular and aural manifestations [64–68]. Ocular findings are inflammatory including anterior uveitis, retinitis, chorioretinitis, retinal vasculitis, and optic neuritis. Ocular and hearing involvement usually has a self-limited course [69, 70], but it can result in persistent impairment [66, 67]. Signs are often subtle and fleeting but persistence of signs has been reported with CHIKV and 5 to 50% of patients may still present prolonged post-infectious rheumatologic complications for several years after the initial infection [71, 72].

Cardiac events have been reported in arbovirus infections with pooled incidence of cardiac events in non-pregnant women of 27.21% for DENV and 32.81% for CHIKV. The highest incidence of dengue-related myocarditis was found in the population younger than 20 years old [73]. However, these events have not been studied in pregnant women and should theoretically be more frequent.

Clinical expression of arboviruses varies between family and genus and within the same genus. In the *Alphaviruses* genus, viruses are classified as either arthritogenic (e.g., CHIKV) or encephalitic (e.g., VEEV) based on their genetic relatedness and the clinical syndromes they cause. This classification can be inappropriate since neurological manifestations have been reported in CHIKV infected adults [74] and newborns [75].

In neonates, the clinical expression of infection resulting from maternal-fetal transmission of arboviruses is different from that of adults and appears to be more difficult to diagnose. It may take the form of respiratory distress, sepsis-like illness, hemodynamic failure, or bleeding syndrome. Potentially fatal complications included meningoencephalitis, myocarditis, seizures, as well as liver and acute respiratory failure [8, 46, 76]. Ocular manifestations were reported after fetal infection with ZIKV [2] and WNV [77]. These complications can lead to neonatal death [76, 78]. Clinicians need to be aware of the clinical features of these infections and they must contemplate the prospect of infection in pregnant voyagers experiencing fever and recent exposure. Vigilance is also necessary regarding the potential for arboviral infections to mimic conditions specific to pregnancy, like HELLP syndrome [79] or eclampsia [62]. Furthermore, asymptomatic infection such as ZIKV can lead to fetal adverse outcomes (e.g., brain and eye defects, microcephaly, and other congenital anomalies) [2] and testing must be done in cases of ultrasonographic findings suggestive of fetal infection first negative checkup.

## Biological testing during pregnancy and Mtct considerations

### Testing for maternal infection

Transient viremia with low viral load and cross-reactivity between flaviviruses are important hurdles that require diagnosis of arbovirus infection to be performed by experienced laboratories. Using clinical symptoms as screening tools is inadequate because arboviral infections can be mild or asymptomatic, and the associated symptoms are non-specific, often overlapping with those of other tropical infections. During pregnancy, it is crucial to make biological confirmation of the infection and to determine the date of onset of symptoms to pinpoint the gestational age at symptom onset. Molecular methods and serology are the two commonly performed techniques for confirming the diagnosis of most arboviruses and for studying MTCT.

PCR testing of blood samples is recommended but limited to the first few days of symptoms (WNV, DENV, CHIKV, and ZIKV). Therefore, a negative RT-PCR evaluation does not rule out the diagnosis of infection. Maternal viremia following arboviral infection is typically brief. However, prolonged ZIKV RNA detection in serum was identified in some ZIKV infected pregnant women up to 46 days after symptom onset and in one asymptomatic pregnant woman 53 days post-exposure [80, 81]. Time-to-loss of ZIKV RNA in pregnant women serum was observed to be 3-fold longer than among nonpregnant women of similar ages [82]. Prolonged viremia after ZIKV infection is associated with fetal adverse outcomes [80, 83] and has not been studied for other arboviruses.

Flavivirus genomes, such as DENV, ZIKV and WNV, can be detected in urine samples for a longer duration compared to serum samples [84]. Since conducting biological testing in areas where arboviruses co-circulate is more challenging [85], it is advisable to preserve both blood and urine samples collected during the early symptomatic phase for pregnant women seeking care at a primary healthcare center. Due to the brief viremia and the restricted access to molecular testing, serological methods, which detect virus-specific IgM antibodies in the serum, are the most used methods for arboviral infections (WNV, CHIKV, YFV).

In case of Flaviviruses infection, seroconversion with IgM positivity suggests recent infection but not very specific, as most infections can induce non-specific elevation of IgM antibodies against arboviruses through cross-reactivity (DENV, ZIKV et and YFV). It must be followed by the plaque reduction neutralization test (PRNT) due to cross-reactivity with other endemic Flaviviruses [86]. However, these procedures remain limited to highly specialized laboratories. If serological testing is negative, particularly in the early phase of infection, it should be repeated one and 2 weeks later. Positive IgG without IgM indicates a past Flavivirus infection without being specific for the type of arbovirale infection. It can stay positive for a long time and cannot be effectively used as a marker of recent infection.

Many arboviruses have been detected breast milk, seminal fluid, saliva, urine, and maternal blood. Only semen and blood products have proved to be infectious. Table 2 provides an overview of documented instances where arboviruses were detected in mother, fetus, and fetal annexes. RNA can be detected in amniotic fluid collected prenatally and /or at birth and in postnatal samples of cord blood, placental tissue, and fetal tissue [87]. Maternal IgG crosses the placenta and can be detected in fetal cord blood (ZIKV, CHIKV, DENV, and WNV).

**Table 2:** Site of detection of arboviruses in maternal, fetal annexes, and in different neonatal samples according to virus type

	Site of detection of RNA		
Virus	Mothers	Fetal annexes	Fetus or neonates
**ZIKV**	Saliva, urine, blood (whole blood, serum, plasma), anal fluid, cervical mucus, vaginal fluid, breast milk, solid organ transplantation, CSF	Amniotic fluid, placenta, membranes, umbilical cord	Cord blood, urine, neonatal blood, CSF, brain
**DENV**	Breast milk, seminal fluid, saliva, urine, blood, solid organ transplantation, CSF	Amniotic fluid, placenta, umbilical cord	Neonatal blood, urine
**JEV**	No data	Placenta	Brain, liver
**WNV**	Blood, serum, urine, brain, CSF, milk	Placenta, umbilical cord	Blood
**YFV**	Blood	No data	Serum samples
**CHIKV**	Breast milk, seminal fluid, saliva, urine, blood, amniotic fluid, placenta, membranes, CSF	Amniotic fluid, placenta	Blood
**VEEV**	Blood, throat swabs, human serum	Amniotic fluid	Brain tissue from aborted and stillborn human fetuses
**OROV**	blood	No data	No data

The identification of the virus at a site does not mean that it can be considered as a possible source of contamination (e.g., ZIKV has been found in breast milk without being a confirmed mode of neonatal contamination)

*CHIKV* Chikungunya virus, *CSF* cerebrospinal fluid, *DENV* Dengue virus, *JEV* Japanese encephalitis virus, *OROV* Oropouche orthobunyavirus, *VEEV* Venezuelan equine encephalitis virus, *WNV* West Nile virus, *YFV* Yellow fever virus, *ZIKV* Zika virus

Until recently, except for ZIKV infection, screening for arbovirus infection during pregnancy relied primarily on maternal symptoms. Fever, joint pain, headaches, with or without skin or mucous lesions, and certain neurological manifestations have been the most reported warning signs that should prompt arboviral infections testing in endemic areas or among pregnant women returning from travel to exposed regions. In areas with active arboviral transmission, co-infections with several arboviruses are not uncommon and must sometimes be investigated and eliminated [16]. The ability to accurately diagnose an arboviral infection is critical for initiating a timely response to infection and to improve pregnancy outcomes. Thus, the communication between the clinician and the biologist is crucial. Like SARS-CoV-2, the development of non-invasive sampling methods, such as saliva tests, should be encouraged.

### Exploration and investigation of mother-to-child transmission (MTCT)

MTCT is crucial to study when arbovirus infections occur during pregnancy. The study of mother-to-child transmission (MTCT) can be initiated during the antenatal period, guided by maternal testing, ultrasound findings, and, when deemed suitable, invasive procedures (e.g., chorionic villus sampling and amniocentesis). MTCT of arbovirus can be considered in several clinical situations. First, in neonates born to mothers with confirmed or suspected infection, arbovirus testing using molecular and/or serologic testing must be enacted at different sampling sites to increase the chances for virus identification. It can include fetal annexes (placenta, membrane, and amniotic fluid), fetal blood, neonatal urine, and cerebrospinal fluid in the symptomatic child. Careful neonatal examination can provide several clues in favor of an antenatal or perinatal infection and must be guided by the clinical context and supported by suitable biological analyses when clinically appropriate.

Secondly, in cases of fetal abnormalities suggestive of fetal infection, screening must include molecular and serological testing in blood, as well as urine and serology testing in the mother. Therefore, ultrasound screenings can contribute to arbovirus diagnostics. Fetal lesions caused by arbovirus infection during pregnancy can manifest as various types of tissue damage detectable by fetal imaging. During the prenatal period, ultrasound findings following infections by arboviruses such as ZIKV and recently VEEV have been well-documented [2, 6]. These abnormalities may include calcifications (brain, liver, bones, eyes), hemorrhagic lesions, necrosis, and edema (accumulation of fluid in tissues), or a fetal immobility sequence [2, 7]. The ultrasound appearance of the lesions varies based on their ages and locations. Placental anomalies are visualized on ultrasound as placental hypertrophy or placental calcifications. Amniocentesis with molecular testing in amniotic fluid must be considered in endemic areas in cases of arboviral infections with ultrasound features suggestive of fetal infection without evident causes. The indication of invasive sampling remains unclear in fetuses without structural antenatal anomalies, especially in ZIKV infection. Molecular diagnosis must be interpreted with caution and the sensitivity of the different available kits must be carefully considered [88]. Molecular identification of arboviruses in amniotic fluid after amniocentesis has been documented for ZIKV and one reported case involving the Tonate virus [6]. The presence of Zika virus RNA in amniotic fluid has shown a significant association with the occurrence of birth defects [4, 87]. In cases where no amniotic fluid sample has been collected during prenatal period, often due to parental refusal of amniocentesis or in unmonitored pregnancies, it is recommended to conduct Zika virus RNA molecular testing on cord blood, umbilical cord tissue, and placental tissue. Additionally, Zika virus RNA testing in amniotic fluid collected at delivery can provide a practical alternative [87].

Finally, in cases of fetal loss (miscarriage and stillbirth), research of a history of fever during pregnancy, flu-like syndrome, and clinical examination must be paired. In this context and in addition to classical testing, molecular testing in placenta biopsy and fetal tissue, placental histology, and autopsy must be considered. Moreover, less invasive examination or alternatives, such as post-mortem high-field magnetic resonance imaging (MRI), might be an acceptable approach to considerer when patient barriers to fetal autopsy exist [89]. We recommend the implementation of a pre-established protocol within maternity units exposed to effectively manage and report infections during pregnancy.

## Impact of Arboviral infections in pregnancy

The relationship between infection and pregnancy can involve three mechanisms: between maternal infection and viremia, placental infection, and fetal infection. Virus and host have a gestational teratogenic window of time during which infection of the brain can result in birth defects (4 to 10 wg). Pregnancy adverse outcomes depend on term of infection, tropism of the virus, and injury caused by the immune response of the host.

### Mechanisms by which some arboviral infections may affect pregnancy

#### More severe infection compared to non-pregnant women

Small studies have suggested that pregnant women seem to be more likely to present more severe forms of dengue infection than the general population [90, 91]. In the study of Machado et al. in Brazil (January 2007 to December 2008), pregnant women had an increased risk of developing severe dengue infection and dying of dengue [90].

#### Severe maternal infection and hemodynamic changes can indirectly affect fetus

Arboviruses can lead to severe maternal infection with dramatic consequence such with DENV, YFV, WNV, and CCHF with an increased risk of maternal mortality [92–94]. DENV increases vascular permeability thereby predisposing one to plasma leakage and has been shown to increase the production of proinflammatory cytokines that can induce uterine activity and potentially initiate preterm labor. Major maternal degradation and hypoxemia can indirectly lead to fetal damage. The observation of massive fetal brain damage secondary to critical cardiopulmonary deterioration and acute maternal hypoxia was also documented in other infections such as with severe SARS-CoV2 [95]. For other arboviruses, despite disease manifestations appearing no more severe than in the general population, arboviruses such as WNV, JEV, and YFV can cause several life-threatening complications [96]. Mortality can result from neuroinvasive disease, as well as hemodynamic and/or visceral failure.

#### Teratogenic effect of arboviral infections

Arboviruses can be teratogenic such as ZIKV and VEEV in the *Flaviviruses* group [6, 97]. Fetal brain development is a dynamic process involving a highly coordinated migration and maturation of neural precursor cells (NPCs) and other brain cells. ZIKV can infect neural progenitors in the developing central nervous system of fetuses. To reach the fetal brain, neurotropic viruses must penetrate the fetal blood-brain barrier. Arboviruses like ZIKV and Tonate virus can infect cortical progenitors [97]. Neurons in the cerebral cortex, thalamus, and hypothalamus had evidence of deterioration [97]. Examination of fetal and neonatal brains infected by ZIKV showed a reduced number of cortical neurons and cortical-layer thickness [97]. On ultrasonographic screening, the range of ultrasonographic and clinical lesions is highly comparable between ZIKV [2] and TONV [6]. The fetus shows severe necrotic and hemorrhagic lesions of the brain and spinal cord [2, 6]. Findings can differ depending upon the time between ultrasound scan and fetal infection. Recent lesions are dominated by necrosis and hemorrhage and calcification appears later. Microcephaly is thought to be the tip of iceberg and shows the defect of the development of the brain. Akinesia and immobilism are the consequence of brain stem injury and can be observed following monitoring of a fetus with amniotic and cerebral infection. Arthrogryposis when present reflects severe lesions [98] and was associated with a 13-fold increased risk of mortality in neonates with congenital Zika syndrome than neonates with congenital Zika syndrome but without arthrogryposis [98].

#### Fetal infection

Once the virus has crossed the placental barrier, fetal lesions depend on the virus’s tropism for fetal tissues, the timing of embryogenesis, the viral cytopathic effects including teratogenic/neurotropic effects, and the associated inflammatory reaction triggered by the production of cytokines/chemokines in fetal tissues. The reported fetal lesions in cases of fetal infection by arboviruses encompass a broad spectrum, including cerebral and extra-cerebral lesions that can ultimately lead to fetal loss.

#### Placental infection and dysfunction

Arboviruses target the fetal-maternal interface resulting in severe placental injury with severe consequence [43, 45]. Some arboviruses can replicate efficiently in maternal placental epithelial cells and in cyto- and syncytiotrophoblasts before infecting fetal tissues [99]. Placental damage following infection is due to infiltration of CD68+ and TCD8+ cells, expression of MMPs, cytokines (IFN-γ and TNF-α), and other immunological mediators (RANTES/CCL5 and VEGFR-2) which cause excessive inflammation and vascular dysfunction resulting in placental dysfunction and reduced maternal-fetal exchanges [100, 101]. These changes are histologically manifested by lesions of chorioamnionitis, infarcts, ischemic necrosis with fibrin deposits, villitis and/or intervillitis, subchorionic thrombosis, calcifications, leukocytic infiltration, and Hofbauer cell hyperplasia. Adverse outcomes such as fetal losses, fetal distress and fetal growth restriction were associated with widespread necrosis of placental tissues associated with severe hemorrhage [99, 102].

### Adverse birth outcomes associated with arboviral infections

**Fig. 1:**
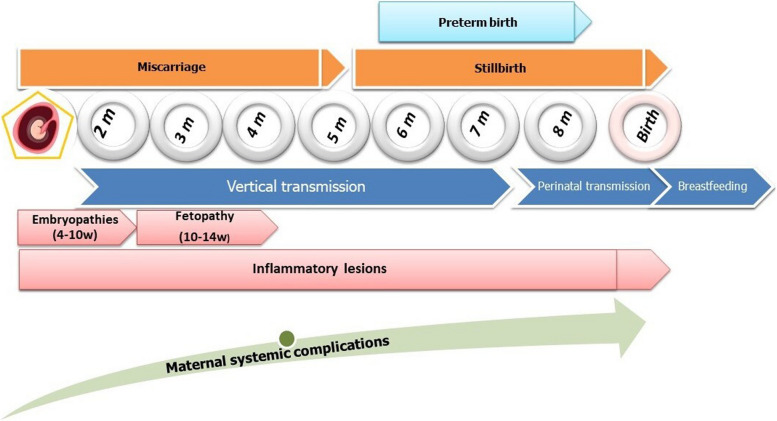
Theoretical framework for arboviral infections exposure risk during pregnancy: analytical findings and maternal-fetal adverse outcomes

Some arboviral infections during pregnancy can be associated with poor pregnancy outcomes such as fetal loss, birth defects, adverse obstetrical outcomes, and neonatal complications [2, 3, 40, 103]. Adverse outcomes depend on several factors: type of arbovirus, the term of pregnancy at the time of infection, teratogenic effect of the virus, placental infection, and maternal systemic and hematologic complications. Figure 1 presents a theoretical framework depicting the risk of exposure, analytical results of arbovirus exposures during pregnancy, and the resulting fetal and maternal adverse outcomes. In endemic areas, many arboviral infections like dengue and Chikungunya may occur during pregnancy without resulting in complications. Adverse outcomes, if present, can vary significantly among different arboviruses.

Firstly, some arboviral infections can result in serious and life-threatening illnesses for pregnant women. Severe maternal complications are more frequent with YFV [92], WNV, and RVFV whereas they are less frequent with DENV [104] and CHIKV infections [94]. No significant association was found between the severity of the maternal infection with DENV and CHIKV and neonatal disease [8].

Secondly, arboviruses can have teratogenic effects, leading to fetal birth defects and long-term sequelae. Teratogenic effects and birth defects are only documented for ZIKV [2, 39, 105] and VEEV [6] infection, and due to the neurotropism to the fetal brain tissue. Among ZIKV-infected pregnant women, vertical transmission occurs in 47% following maternal infection in the first trimester, 28% in the second, and 25% in the third trimester [39]. Among them, congenital Zika syndrome will occur in 9, 3, and 1%, respectively [39] and microcephaly in 2%. Birth defects appear to be primarily associated with infections that occur during the first trimester [39]. The percentage of vertical transmission for VEEV infection remains unknown.

Thirdly, adverse obstetrical complications, such as fetal distress during labor, peripartum hemorrhage, preterm birth and emergency cesarean sections is mainly associated with dengue with warning signs [40, 103]. Obstetric hemorrhage can occur following dengue infection with warning signs in 22% of cases [40] and in up to 30% of severe dengue cases [103]. Low birthweight and acute fetal distress during labor are associated with arboviruses such as DENV [40, 106]. In addition, some arboviral infections can lead to miscarriage and fetal losses. These outcomes are reported after Flavivirus infections like DENV [40, 106–108], ZIKV [2, 3] and VEEV, Alphavirus infection such as CHIKV, and after others arboviruses like RVFV [109, 110] and JEV [111].

Fourthly, some arboviral infections can result in perinatal transmission with potential risk of complication in neonates. Severe complications are mostly reported with CHIKV [42, 112], YFV [76], and to a lesser extent, in cases of DENV [106], ZIKV [113], WNV [54], and RVFV [114, 115]. They can be septic, hemorrhagic, or neurological and can result in neonatal death [75, 112, 114]. Neonatal adverse outcomes following arbovirus infection can exhibit variations across different regions. For instance, during a CHIKV epidemic in the southern part of Reunion Island, severe manifestations like meningoencephalitis, intravascular coagulations, and severe intracerebral hemorrhage were reported [46], These findings resembled those observed during the CHIKV outbreak on Curaçao Island [75]. In India and French Guiana, clinical presentations included apnea, fever, erythematous maculo-papular rash, and generalized hyperpigmentation; however, no neurological complications were documented. Variations in outcomes may stem from factors such as genotype, genetic exposure in parturient individuals, co-infections, or unidentified protective factors.

**Fig. 2:**
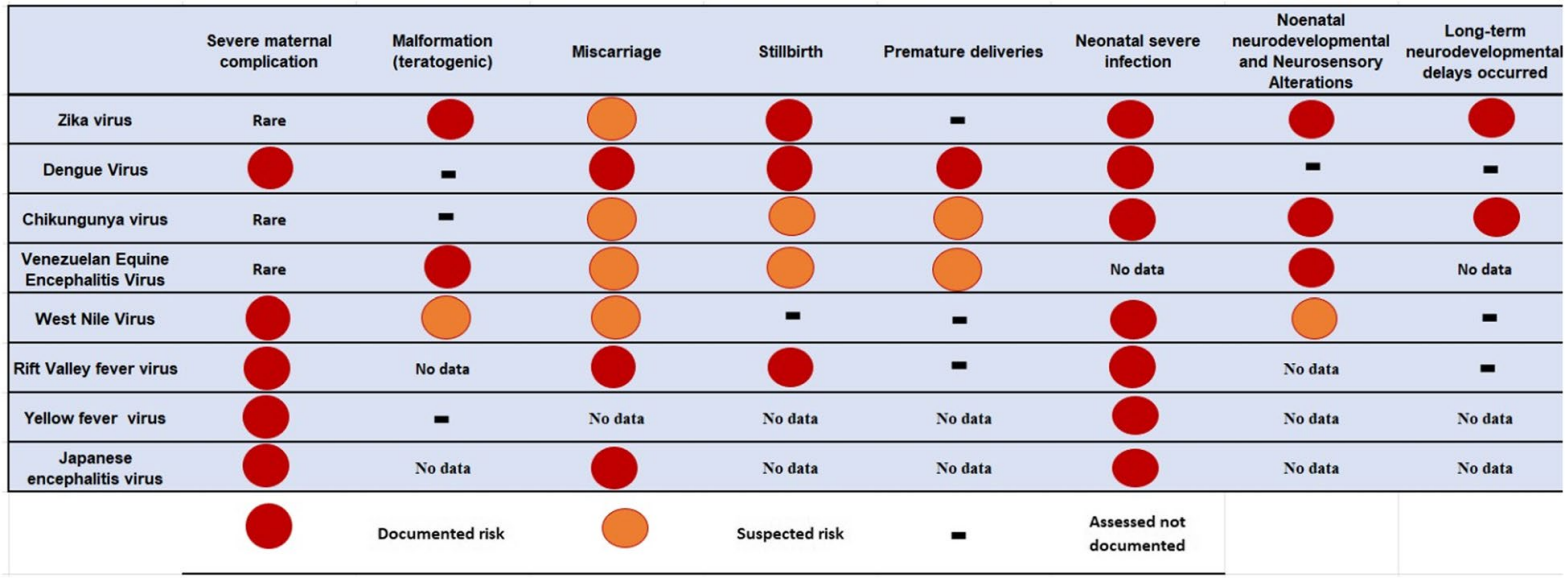
Fetal and neonatal consequences of arboviral infections during pregnancy

Finally, neurological damage caused by teratogenic arboviruses can ultimately result in long-term neurosensory delays and consequences [4, 75] particularly after infection during the first trimester [39]. ZIKV infection is well-documented to lead to neurosensory alterations, including visual and hearing impairments [116], delayed childhood neurodevelopment, and cognitive deficits [4, 116]. In the Reunion Island outbreak, CHIKV infection also showed a tendency towards such consequences, though to a lesser extent [46]. These long-term effects were confirmed at 2 months [4], and during the second and third years of life [4, 116]. ZIKV infected fetus with normal evaluation at birth may develop postnatal microcephaly and/or subsequent neurodevelopmental disorders [116]. Autism spectrum disorders have also been reported [116]. Infected neonates without congenital Zika syndrome also appear to be at risk, although to a lesser extent, for developmental delay [4, 116]. Figure 2 provides an overview of the adverse outcomes, both maternal and neonatal, that can be associated with arbovirus infection during pregnancy.

## Prevention and perspectives

Currently, only vaccines against YFV and JEV are readily accessible and effective. YFV and JEV vaccines have been given to many pregnant women without any apparent adverse effects on the fetus, but their administration is still recommended to be deferred until after pregnancy [117, 118]. The treatment for arbovirus symptomatic infection remains supportive with no effective therapy. Using tocolytics in cases of at-term perinatal infection with risk of neonatal transmission (e.g., CHIKV and YFV) was not evaluated. Protection against mosquito bites should be encouraged by wearing protective clothing and using insect repellents. Often during pregnancy, people are reluctant to use repellent, despite it being safe and still recommended. Additionally, this approach can be applied more broadly to the prevention of other vector-borne diseases in pregnant women, such as malaria. Two periods are critical: first trimester of pregnancy and close to the date of delivery. This can also involve removal of mosquito breeding sites and mosquito nets. During an outbreak, the control of mosquitos in the hospital setting should also be addressed [119].

Predicting when and where arbovirus infection outbreaks will occur is challenging. Bio-surveillance in areas with high infectious potential and bacteriological surveillance study of insects [120] can help to identify mosquito vectors and reservoirs. Combining real-world data and big data is a promising approach to improve arbovirus prediction and monitoring. The most significant predictors have been reported to be rainfall (43%), temperature (41%), and humidity (25%) [120]. Progress is needed to develop more efficient, affordable, sensitive, and time-saving techniques for detecting most arboviruses. The interconnection between the health of humans, animals, and their shared environment emphasizes the need for collaboration between health authorities, veterinary, entomologists, environmental specialists, and agricultural authorities as the best strategy detecting and responding to emergent arboviral epidemics.

Artificial intelligence can utilize environmental and climate data to predict disease outbreaks. The use of a smartphone app in an arbovirus-endemic region can contribute to surveillance and diagnosis of arbovirus infections in pregnancy [121].

## Conclusion

This review highlights the real threat from arboviral disease to both the mother and fetus. In view of climate change, urbanization, travel, and immigration, lesser and well-known arboviruses have the potential to spread geographically. Beyond their effect on the mother’s health, some arboviral infections can lead to fetal loss, obstetrical complications, neonatal infection, and can be teratogenic with long-term neurological disorders and impaired visual and hearing function. The actual burden of arbovirus infection and real incidence of adverse fetal outcomes during pregnancy remains under-reported. Particular attention must be given to pregnant women and their partners who travel to endemic areas. Additional extensive cohort studies involving pregnant women are essential to assess whether lesser-known arbovirus infections also contribute to adverse pregnancy outcomes. There is an urgent need to further develop research, effective surveillance, diagnostics, and therapies.

## Data Availability

Data and material availability: the data presented in this study are drawn from publicly available sources and previously published works, which are cited accordingly. A list of articles is available on request.
